# Promoting early presentation of breast cancer in older women: sustained effect of an intervention to promote breast cancer awareness in routine clinical practice

**DOI:** 10.1186/s12913-017-2335-8

**Published:** 2017-06-05

**Authors:** Rachael H. Dodd, Alice S. Forster, Sarah Sellars, Julietta Patnick, Amanda J. Ramirez, Lindsay J. L. Forbes

**Affiliations:** 1 King’s College London, Research Oncology, 3rd floor Bermondsey Wing, Great Maze Pond, London, SE1 9RT UK; 2 NHS Cancer Screening Programmes, Fulwood House, Old Fulwood Road, Sheffield, S10 3TH UK; 3 University of Sydney, School of Public Health, Edward Ford Building, Sydney, NSW 2006 Australia

**Keywords:** Breast cancer, Intervention, Awareness, Symptoms, Health professionals, Clinical practice

## Abstract

**Background:**

Older women have poorer survival from breast cancer, which may be at least partly due to poor breast cancer awareness leading to delayed presentation and more advanced stage at diagnosis. In a randomised trial, an intervention to promote early presentation of breast cancer in older women increased breast cancer awareness at 1 year compared with usual care (24 versus 4%). We examined its effectiveness in routine clinical practice.

**Methods:**

We piloted the intervention delivered by practising health professionals to women aged about 70 in four breast screening services. We measured the effect on breast cancer awareness at 1 year compared with comparison services, where women did not receive the intervention.

**Results:**

At 1 year, 25% of women in pilot services were breast cancer aware compared with 4% in comparison services (*p* = 0.001). The components of breast cancer awareness were knowledge of breast cancer non-lump symptoms (pilot: 63% vs comparison: 82% at 1 year; OR = 2.56, 95% CI 1.92-3.42), knowledge of age related risk (pilot: 8% vs comparison: 36% at 1 year; OR = 5.56, 95% CI 4.0-7.74) and reported breast checking (pilot: 70% vs comparison: 78% at 1 year; OR = 1.49, 95% CI 1.13-1.96).

**Conclusion:**

The intervention may be as effective in routine clinical practice as in a randomised controlled trial. This intervention has the potential to reduce patient delay in the diagnosis of breast cancer in older women.

**Trial registration:**

The PEP trial was registered with the International Standard Registered Clinical/soCial sTudy Number (ISRCTN) as a clinical trial (ISRCTN31994827) on 3rd October 2007.

## Background

Women in the United Kingdom (UK) have poorer survival from breast cancer compared to other similar countries [1, 2]. While this may be due to delays in diagnosis or treatment once in the health care system, or less aggressive treatment once in the health care system, patients presenting when breast cancer has already reached a late stage may also contribute. Delayed presentation of breast cancer is more common in older than younger women [3] and older women are more likely to be diagnosed with more advanced disease [4]. Not recognising breast cancer symptoms is a risk factor for delayed presentation [3, 5]. Improving symptom recognition to facilitate prompt presentation in this population may improve survival.

The Promoting Early Presentation (PEP) Intervention was developed to provide older women with the knowledge, motivation, confidence and skills to present promptly on discovering a breast symptom. It is a brief, scripted, one-to-one intervention delivered by a health professional in a positive, motivational and collaborative style and supported by a booklet [6, 7]. In a randomised controlled trial (RCT), the PEP Intervention delivered by research radiographers, increased breast cancer awareness in women around the age of 70 compared with usual care after 1 year (breast cancer aware: PEP Intervention 24% vs usual care 4%) [7]. In a pilot in the National Health Service (NHS) Breast Screening Programme, in which the PEP Intervention was delivered as part of routine clinical practice by NHS radiographers to women of a similar age, breast cancer awareness increased from 4% before the intervention to 38% 1 month later [8]. We aimed to measure whether this effect was maintained after 1 year to the same extent as in the trial.

## Methods

Women attending for their final invited screening appointment at four NHS breast screening services where the PEP Intervention was offered (pilot services) and at a comparison service where the PEP Intervention was not offered (comparison service) were invited to take part during May 2011 and April 2012. Recruitment is described in more detail elsewhere [8] and informed consent was obtained from all participants.

Women completed a questionnaire before their mammogram. The questionnaire included a validated measure of breast cancer awareness [9]. Women were considered breast cancer aware if they recognised five or more non-lump symptoms (change in the position of nipple, pulling in of nipple, pain in breasts or armpit, puckering or dimpling of breast skin, discharge or bleeding from nipple, nipple rash, redness of breast skin, changes in the size of breast or nipple, changes in the shape of breast or nipple), knew that the risk of breast cancer increased with age and reported checking their breasts at least once a month. The questionnaire also asked for details of ethnic group, whether they lived with a husband or partner and the age they had left full-time education. At 1 year, the women were sent a further questionnaire to measure breast cancer awareness.

We excluded from the analysis women who reported being treated for breast cancer during that year. We included only women who had provided data at baseline and 1 year on all three relevant questions. We used women’s responses to the three breast cancer awareness questions to calculate a breast cancer awareness score (range 0-3). Women were considered breast cancer aware if they scored 3 out of 3, meaning that: they knew that risk of breast cancer increases with age (knowledge of age-related risk), could identify five or more non-lump symptoms of breast cancer (knowledge of non-lump symptoms) and reported checking their breasts at least once a month.

To examine change in breast cancer awareness from baseline to 1 year, we used repeated measures logistic regression models. These logistic regression models (or generalised estimated equations) allow us to model the relationship between covariates of interest (e.g. receiving PEP Intervention or not, time) and an outcome (breast cancer awareness) measured at multiple time points, accounting for the correlated nature of the data. These calculated odd ratios (ORs) with 95% confidence intervals (CI) for odds of being breast cancer aware (i.e. scoring 3 in the breast cancer awareness score) in women who received the PEP Intervention compared to those in the comparison service. We also carried our similar analyses for the three component questions of the breast cancer awareness score: for the odds of knowing the age-related risk of breast cancer and non-lump symptoms, and for breast checking at least once a month. We examined the effect on the odds ratios of controlling for differences in age, whether they had a husband/partner, age at leaving full-time education and Index of Multiple Deprivation (IMD), a score based on area of residence using data from a number of sources, which provides an estimate of socioeconomic deprivation.

## Results

In the four pilot breast screening services, 356 women provided analysable breast cancer awareness data at 1 year, representing 72% of the 497 who completed the baseline questionnaire (2 had been diagnosed with breast cancer, 93 did not respond at 1 year, and 46 did not complete all three relevant questions). Details of non-responders at 1 month are given in our previous paper [8]. Those excluded from the analysis were more likely to be living in socioeconomically deprived areas and less likely to be breast cancer aware at baseline than those who were included.

In the comparison services, 661 women provided analysable breast cancer awareness data, representing 75% of the 880 who completed the baseline questionnaire (4 had been diagnosed with breast cancer, 154 did not respond at 1 year, and 61 did not complete all three relevant questions). We found no demographic differences between those included in the analysis and those not, although women who were excluded were less likely to be breast cancer aware at baseline.

There were some demographic differences between women in the pilot and comparison services: women in the pilot services were slightly older, less likely to be living with a husband or partner, less socioeconomically deprived and more educated (described in more detail in our previous paper [8]).

Women who received the PEP Intervention were significantly more likely to be breast cancer aware at 1 year than women in the comparison service (25 vs. 4%; Table 1). This was true for all components of the breast cancer awareness score, the effect being greatest for knowledge that the risk of breast cancer increases with age (Table 1). Adjusting for demographic differences (age, living with a husband or partner, age left full-time education and IMD score) between the groups made little difference to the odds ratios (Table 1).

**Table 1:** Adjusted and unadjusted results for breast cancer awareness at baseline and 1 year

	Comparison service (*n* = 661)		PEP Intervention service (*n* = 356)		Odds ratio (PEP Intervention vs comparison service (95% CI, *p* value)	
	Baseline	One year	Baseline	One year	Unadjusted	Adjusted^b^
Breast cancer awareness^a^ Number (%) breast cancer aware	22 (3.3)	29 (4.4)	19 (5.3)	90 (25.3)	6.97 (4.56-10.63), <0.001	8.13 (4.30-15.33), <0.001
Knowledge of breast cancer symptoms Number (%) who identified five or more non-lump symptoms	374 (56.6)	418 (63.2)	198 (55.6)	291 (81.7)	2.56 (1.92-3.42), <0.001	2.54 (1.72-3.75), <0.001
Knowledge of age-related risk Number (%) who identified that a 70 year old woman is more at risk of breast cancer than a younger woman or woman of any age	57 (8.6)	55 (8.3)	52 (14.6)	129 (36.2)	5.56 (4.0-7.74), <0.001	5.38 (3.28-8.79), <0.001
Breast checking Number (%) who reported checking their breasts at least once a month	384 (58.1)	460 (69.6)	184 (51.7)	277 (77.8)	1.49 (1.13-1.96), 0.005	1.74 (1.18-2.56), 0.005

*Abbreviation*: *CI* Confidence Intervals

^a^A woman scored three points on the breast cancer awareness score (and so was considered to be breast cancer aware) if she: identified at least five non-lump symptoms of breast cancer (one point), identified that a 70 year old woman is most at risk of breast cancer (one point) and reported checking her breasts at least once a month (one point)

^b^Adjusted for age, living with a husband or partner, age left full-time education and Index of Multiple Deprivation score

## Discussion

An intervention to promote early presentation of breast cancer among older women delivered by NHS mammographers in routine clinical practice raises breast cancer awareness to a similar extent as in a research setting. All three components of breast cancer awareness were increased in women receiving the PEP Intervention when compared to the comparison service at 1 year. The most marked effect was seen in women’s knowledge of age-related risk, albeit knowledge was still much lower than for the other components of breast cancer awareness.

The intervention was conducted at the final scheduled mammography appointment, so it is surprising that the level of awareness in women was in fact not higher than found. However, previous studies with older women have also found low levels of breast cancer awareness [10]. An explanation for this could be the role of subjective norm, being advice from friends, family and health professionals about mammography, which has been shown previously to influence breast screening uptake [11]. This could lead women to feel less engaged with their decision and therefore be less breast cancer aware. Alternatively, the women who attend screening may be those who do have poor knowledge of breast symptoms, personal risk and feel less confident about detecting a breast change and this might be their reason for attending screening.

Similar increases in magnitude for women’s knowledge of age-related risk were found in the implementation of the PEP Intervention into routine clinical practice to those found in the RCT [7], which is very encouraging. Although the overall level of knowledge is lower than the other two components, the level of knowledge from baseline has improved greatly. Previously, women have expressed surprise that their risk of developing cancer increased with age, but that they would no longer be invited for mammograms [6]. As no more invitations for screening can lead women to infer that risk diminishes with age, this is likely to be a reason for this component of breast cancer awareness being the most difficult to achieve high levels of awareness. In addition, modelling and demonstration for breast checking and non-lump symptoms may have reinforced those messages and resulted in those two components being more potent. It is important to communicate the message to older women that risk increases with age when they interact with a health professional.

The high response rates and use of a validated measure of breast cancer awareness [9] are strengths of this study. We found no evidence that the increase in breast cancer awareness was due to the questionnaire measuring breast cancer awareness itself (an effect known as the ‘mere measurement effect’ [12]) because there was only a very small increase in breast cancer awareness in the comparison service.

There were demographic differences between women in the pilot services and the comparison service. However, we think this is unlikely to explain our findings – controlling for this made little difference to the magnitude of the effect.

We carried out the analysis only on those women with complete data at 1 year. The women who were excluded from this were less likely to have been breast cancer aware at baseline. This could have spuriously inflated differences in breast cancer awareness at 1 year between PEP Intervention and comparison services, but the differences are so striking that it is very unlikely to explain them fully.

In a 2013 report, the All Party Parliamentary Group on Breast Cancer recommended that the PEP Intervention be more widely implemented so that the effect on breast cancer mortality can be evaluated [13]. One- to-one interventions such as the PEP Intervention and public awareness campaigns such as the national Be Clear on Cancer campaign to promote early presentation in women aged 70+ with breast symptoms [14] may ultimately lead to improving the UK’s poor breast cancer survival compared with other high income countries with similar health care systems. It is currently unknown whether increased breast awareness, and what level of awareness, will result in reduced breast cancer mortality. However, given that women with poor awareness of symptoms have been shown to delay presentation [3], this could suggest an influence of breast awareness in mortality. Self-referral for screening, symptomatic breast clinic attendances and breast cancer mortality will be monitored in this group of women in the longer term.

The success of the intervention in a mammography setting has led to further adaptation of the intervention to enable it to be transferable to other settings such as general practice [15], to additionally target those who do not attend for screening. Implemented into general practice by practice nurses, breast cancer awareness increased between baseline and 1 year by a similar magnitude found in the present study [15]. Cost effective alternatives of the PEP Intervention which have been shown to increase breast cancer awareness, but not to the same magnitude, include a written version of the intervention [16] and also the booklet which supported the PEP Intervention [7]. These could potentially be used to reach greater numbers of older women.

## Conclusion

Women who received the PEP intervention in routine clinical practice demonstrated similar levels of breast cancer awareness at 1 year as those women receiving the PEP intervention in a randomised controlled trial. Follow up showing the effect of this kind of intervention in routine clinical practice after 1 year is unprecedented. These findings can usefully inform future interventions aimed at promoting the early presentation of breast cancer in older women.

## Abbreviations

IMD: Index of Multiple Deprivation
NHS: National Health Service
PEP: Promoting Early Presentation
RCT: Randomised controlled trial
UK: United Kingdom
